# Complete Metabolic Remission With Dose Frequency-Modified Tepotinib Following Capmatinib Intolerance in MET Exon 14-Mutated Non-small Cell Lung Cancer (NSCLC): A Case Report

**DOI:** 10.7759/cureus.117711

**Published:** 2026-10-09

**Authors:** Sanchayan Mandal

**Affiliations:** 1 Oncology, HCG Cancer Centre, Kolkata, IND

**Keywords:** capmatinib, complete remission, met exon 14, nsclc, peripheral edema, sequential met tki, tepotinib

## Abstract

MET exon 14 (METex14) skipping mutations occur in a subset of non-small cell lung cancer (NSCLC) cases, mostly affecting older patients with multiple comorbidities. Selective MET tyrosine kinase inhibitors (TKIs) capmatinib and tepotinib represent the standard of care; however, real-world management is frequently complicated by agent-specific toxicity and class-effect peripheral edema. Sequential use of MET TKIs following agent-specific intolerance and the feasibility of non-standard tepotinib dosing schedules remain poorly characterized. A 74-year-old man with diabetes mellitus, hypertension, and stage IVA METex14-mutated lung adenocarcinoma (cT3N3M1a, malignant pleural effusion, KRAS p.G12V co-mutation) received three cycles of first-line pemetrexed plus carboplatin with residual active disease. Capmatinib 200 mg twice daily, initiated as biomarker-directed therapy, was discontinued after 1.5 months due to severe multi-organ toxicity, including grade 3 hyperglycemia, acute kidney injury, elevated pancreatic enzymes, hypercalcemia, and hyponatremia, with Eastern Cooperative Oncology Group (ECOG) performance status worsening from 2 to 3. Tepotinib 225 mg once daily was initiated with early clinical and biochemical improvement. Grade 3 bilateral pedal edema at two months necessitated stepwise dose-frequency modification of tepotinib together with diuretic therapy. Apparent radiological progression at 10 months was managed as suspected pseudoprogression in view of sustained clinical stability, and therapy was continued. After 13 months of tepotinib therapy, 18F-fluorodeoxyglucose (FDG) positron emission tomography (PET)-computed tomography (CT) demonstrated complete metabolic remission in February 2024. This case shows that capmatinib intolerance does not prevent the benefit from sequential tepotinib and that adjusting the dose frequency can effectively manage MET inhibitor-associated peripheral edema without compromising oncological outcomes, achieving complete remission in an elderly comorbid patient.

## Introduction

Non-small cell lung cancer (NSCLC) accounts for approximately 85% of all lung cancer diagnoses globally and continues to be the primary cause of cancer-related mortality worldwide [1]. Among these molecular alterations, MET exon 14 (METex14) skipping mutations represent a clinically significant and actionable subset, occurring in approximately 3-4% of NSCLC cases [2]. These mutations result in loss of the juxtamembrane domain of the MET receptor tyrosine kinase, impairing ubiquitination and degradation, thereby leading to sustained oncogenic MET signaling [3]. A defining epidemiologic feature of METex14-mutated NSCLC is its strong association with advanced age, with patients typically 70 years of age or older frequently presenting with multiple comorbidities that complicate therapeutic decision-making [3]. The selective MET tyrosine kinase inhibitors (TKIs) capmatinib and tepotinib were approved on the basis of the GEOMETRY mono-1 and VISION trials, respectively [3,4]. Both agents are listed as the preferred first-line and subsequent-line options by the American Society of Clinical Oncology (ASCO) and National Comprehensive Cancer Network (NCCN) guidelines [5].

Despite comparable efficacy, peripheral edema is the hallmark class-effect toxicity of MET inhibitors, reported in 47-51% of patients receiving capmatinib and 63-70% of patients receiving tepotinib, with grade 3 or higher events occurring in 8-11% of patients [6]. The management of peripheral edema in elderly patients with pre-existing comorbidities presents a particularly complex clinical challenge [7], and real-world data on dose-frequency modification approaches and their oncological impact remain scarce. Importantly, switching between MET TKIs because of intolerance is clinically distinct from switching for disease progression, as agent-specific intolerance does not imply resistance to MET inhibition. Published experience with sequential MET TKI use after intolerance is limited to isolated case reports, including successful tepotinib treatment without toxicity recurrence after capmatinib-induced interstitial lung disease or pneumonitis [8,9] and, conversely, continuation of MET inhibition by switching from tepotinib to capmatinib in a patient with intolerable tepotinib-induced peripheral edema [10]. We present a case of an elderly, comorbid patient with advanced METex14-positive NSCLC who achieved complete metabolic remission on third-line tepotinib through a stepwise dose-frequency modification strategy following capmatinib intolerance.

## Case presentation

A 74-year-old man with diabetes mellitus and hypertension presented in September 2022 with progressive respiratory distress, shortness of breath, hemoptysis, right-sided chest pain, and significant weight loss. His Eastern Cooperative Oncology Group (ECOG) performance status was 2. Pleural fluid cytology verified adenocarcinoma. Magnetic resonance imaging (MRI) of the brain was negative for intracranial disease. Molecular profiling of the baseline tissue biopsy specimen by a next-generation sequencing (NGS) panel (Triesta Sciences, Bengaluru, Karnataka, India) revealed a KRAS p.G12V mutation with a PD-L1 tumor proportion score of 3%, together with a METex14 skipping mutation identified on the same panel; variant allele frequencies were not available from the laboratory report (Figure 1). 

**Figure 1:**
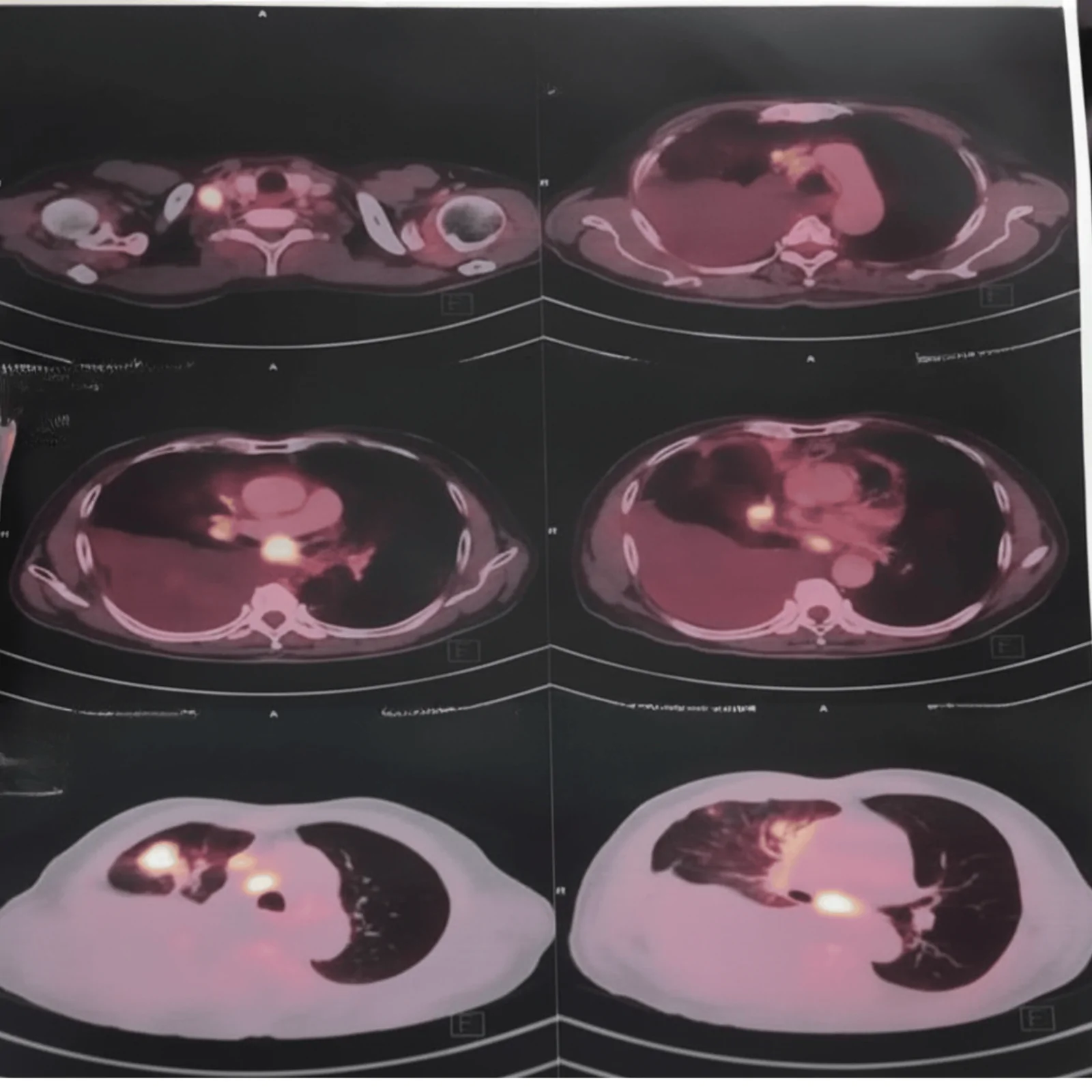
Baseline 18F-fluorodeoxyglucose (FDG) positron emission tomography (PET)-computed tomography (CT) scan performed in September 2022, obtained prior to the initiation of treatment for initial disease assessment

A whole-body 18F-fluorodeoxyglucose (FDG) positron emission tomography (PET)-computed tomography (CT) scan conducted on the same date demonstrated a metabolically active lesion in the right upper lobe with ipsilateral satellite nodules, hilar-mediastinal and right lower cervical lymphadenopathy, and malignant right pleural effusion with no distant metastases. According to the Ninth Edition of the American Joint Committee on Cancer (AJCC)/Union for International Cancer Control (UICC) Tumor, Node, Metastasis (TNM) classification, the disease was staged cT3N3M1a, consistent with stage IVA lung adenocarcinoma: T3 reflecting primary tumor size (5.7×5.5×4.3 cm), N3 reflecting ipsilateral supraclavicular nodal involvement in addition to hilar-mediastinal disease, and M1a reflecting the malignant pleural effusion.

First-line systemic therapy was initiated with pemetrexed plus carboplatin. Between September and November 2022, he received three cycles of pemetrexed 750 mg plus carboplatin 380 mg (cycles 1 and 2), reduced to pemetrexed 700 mg plus carboplatin 370 mg at cycle 3 following an intercurrent fungal pneumonia after the first cycle. During treatment, renal function was evaluated, showing an estimated glomerular filtration rate (eGFR) of 75 mL/min/1.73 m² and a serum creatinine level of 1.02 mg/dL, confirming adequate renal reserve throughout the chemotherapy course. In December 2022, a restaging FDG PET-CT showed objective radiological response to chemotherapy, with significant reduction in primary lesion metabolic activity and near-complete resolution of pleural effusion and nodal disease (Figure 2). By the Response Evaluation Criteria in Solid Tumors (RECIST) 1.1 criteria, this corresponded to a partial response (formal target-lesion measurements were not available; the response was assessed qualitatively on serial FDG PET-CT, and partial response was confirmed by the treating clinician). Chemotherapy was not continued beyond three cycles owing to treatment-related toxicity and the patient's reluctance to receive further cytotoxic therapy. Because baseline NGS had identified a METex14 skipping mutation and residual metabolically active disease persisted, treatment was changed to MET-directed therapy. This was a biomarker-directed switch made in the absence of documented disease progression, rather than a change mandated by progression. In December 2022, the patient was initiated on second-line capmatinib 200 mg orally twice daily. After around 1.5 months of capmatinib therapy, severe multi-organ toxicity was documented: blood glucose 350 mg/dL, serum creatinine 1.5 mg/dL, markedly elevated amylase and lipase, hypercalcemia, hyponatremia, worsening dyspnea, and 2 kg weight loss. ECOG deteriorated from 2 to 3. Capmatinib was stopped in approximately February 2023.

**Figure 2:**
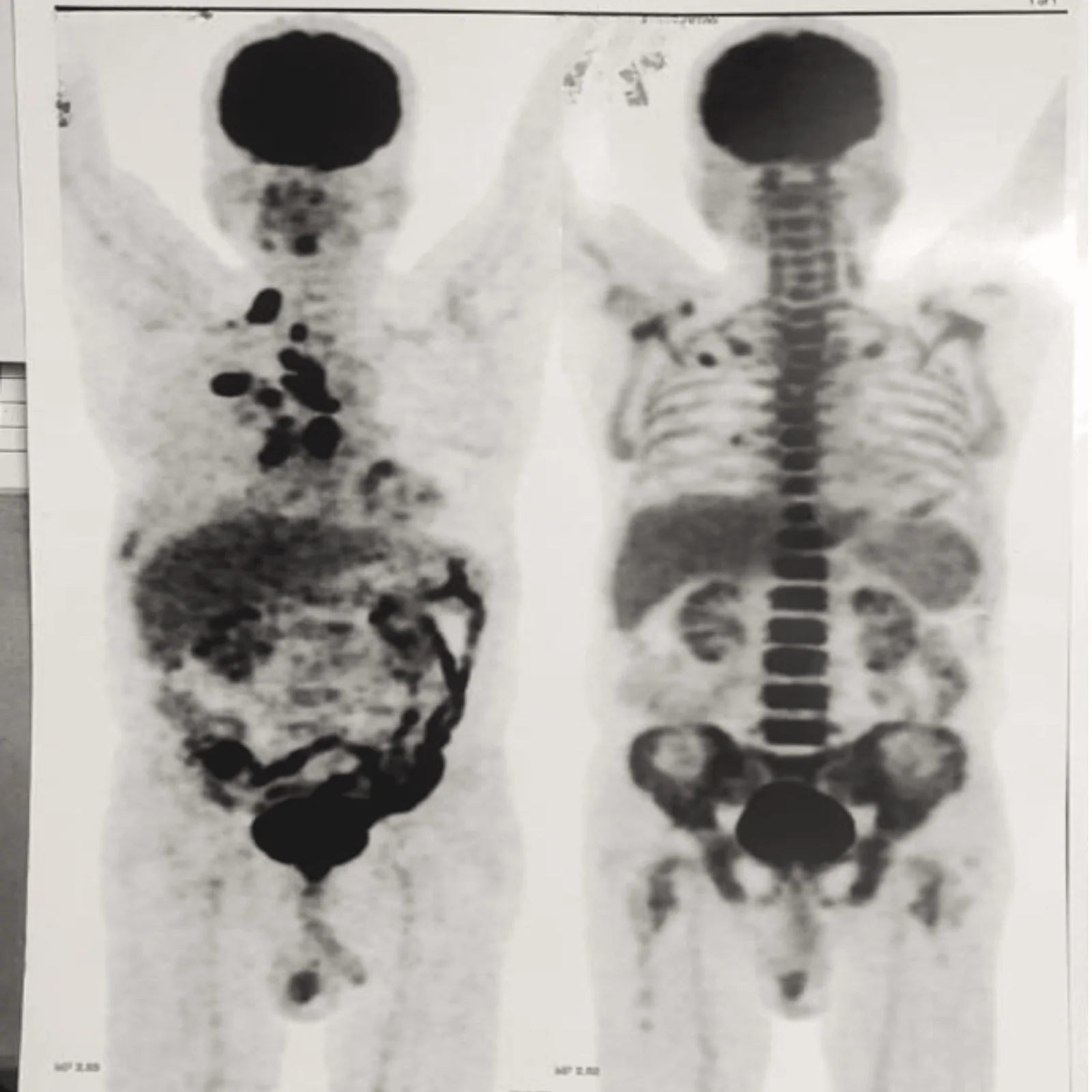
Restaging 18F-fluorodeoxyglucose (FDG) positron emission tomography (PET)-computed tomography (CT) performed in December 2022 to evaluate treatment response and current disease status

Tepotinib 225 mg orally once daily was initiated in February 2023, at half the labelled 450 mg starting dose in view of the prior toxicity to both chemotherapy and capmatinib. Within two months, biochemical parameters returned to normal, respiratory symptoms resolved, and functional status improved.

In April 2023, bilateral grade 3 pedal edema was found on clinical examination (Figure 3). Bilateral lower limb venous Doppler ultrasonography was conducted, and deep venous thrombosis was excluded. The administration of tepotinib was changed from daily to every other day, and 5 mg of torsemide was added twice a day. By May 2023, edema improved to grade 2, though residual swelling persisted; accordingly, torsemide was increased to 20 mg daily. Tepotinib schedule was again changed to a Monday-Friday routine, five days per week with a structured weekend holiday, and a calcium-restricted diet was simultaneously instituted. Weight and edema improved progressively. Restaging FDG PET-CT in June 2023, compared with the December 2022 scan, showed marginal reduction in primary lesion metabolic activity, significant nodal regression, and complete pleural effusion resolution. A subsequent October 2023 FDG PET-CT showed an apparent increase in radiological burden (increased mediastinal nodal uptake with regression at other disease sites). In view of sustained clinical and biochemical stability, this was managed as suspected pseudoprogression, and tepotinib was continued beyond apparent radiological progression with close clinical monitoring. In February 2024, after approximately 13 months of continuous tepotinib, the patient reported fatigue, anorexia, mild weight loss, and a serum creatinine of 1.7 mg/dL. Tepotinib was subsequently reduced to 225 mg three days per week (Monday, Wednesday, Friday), with torsemide reduced back to 10 mg daily as edema remained well controlled, and definitive FDG PET‑CT in February 2024 confirmed complete metabolic remission (Figure 4).

**Figure 3:**
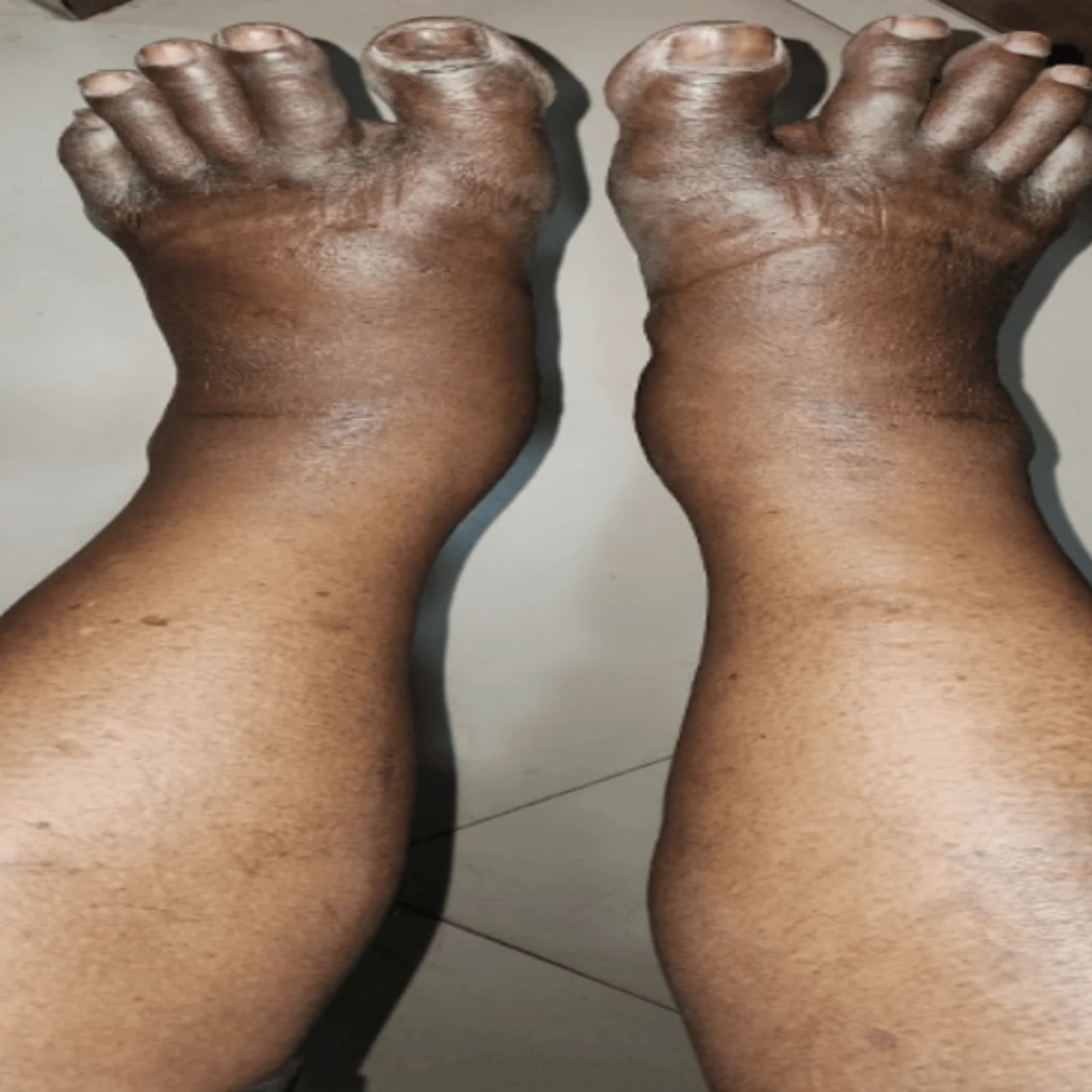
Pedal edema observed in April 2023, indicating swelling of the feet and/or ankles

**Figure 4:**
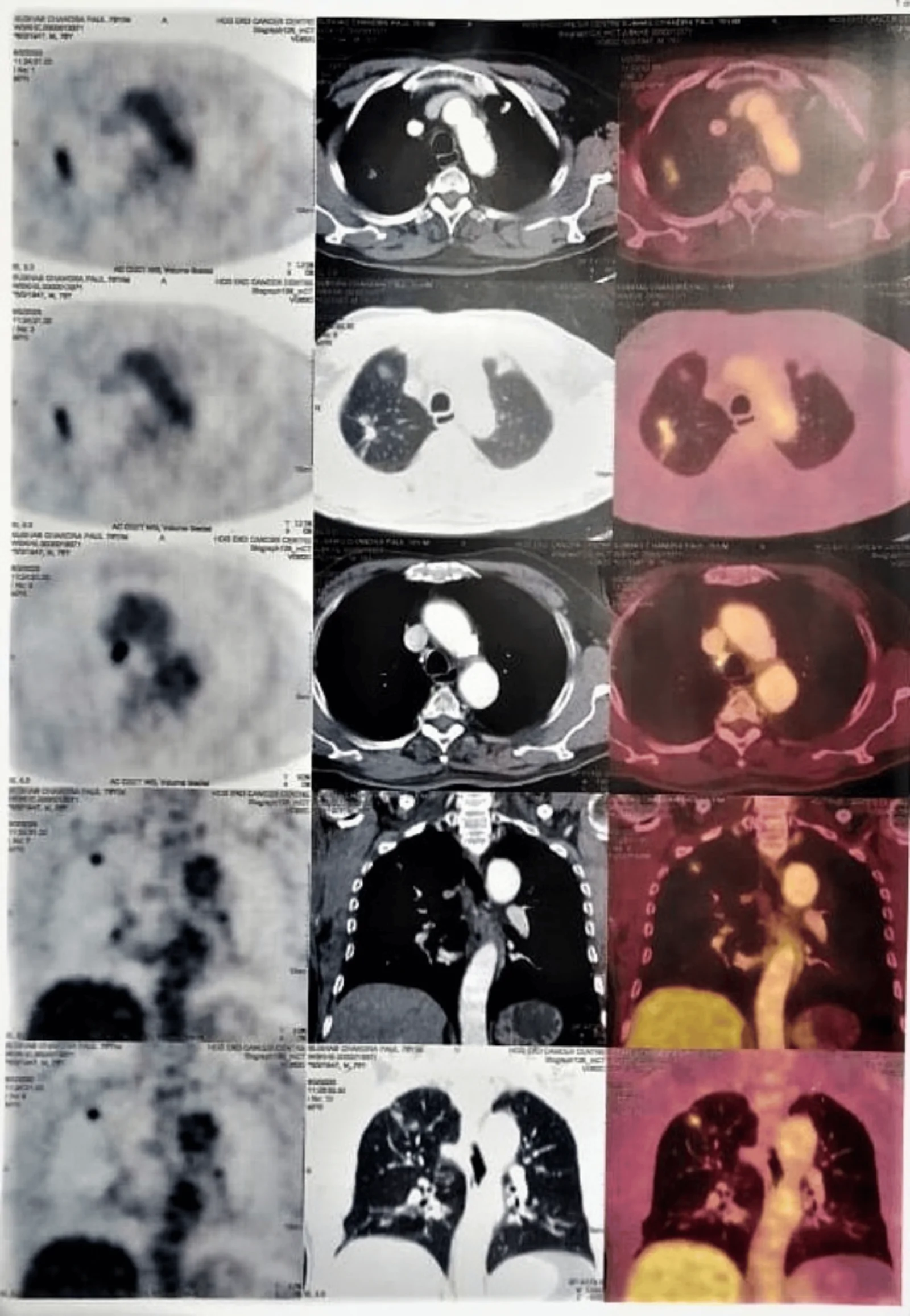
Restaging 18F-fluorodeoxyglucose (FDG) positron emission tomography (PET)-computed tomography (CT) performed in February 2024, demonstrating complete metabolic remission with no evidence of active residual or recurrent disease

The complete clinical timeline is summarized in Table 1.

**Table 1:** Clinical timeline of disease course, treatment sequence, response assessments, and adverse event management ECOG: Eastern Cooperative Oncology Group; PS: performance status; PD‑L1: programmed death‑ligand 1; MRI: magnetic resonance imaging; eGFR: estimated glomerular filtration rate; BD: twice daily; OD: once daily; DVT: deep venous thrombosis: USS: ultrasonography; FDG: 18F-fluorodeoxyglucose; PET: positron emission tomography; CT: computed tomography; RUL: right upper lobe

Timeline	Event/treatment	Response/findings	Notes
September 2022	Presentation + FDG PET-CT (baseline)	Metabolically active RUL mass; hilar-mediastinal + right lower cervical lymphadenopathy; malignant right pleural effusion; no distant metastasis beyond the pleura; right lower neck node confirmed ipsilateral supraclavicular (cT3N3M1a, stage IVA; primary 5.7×5.5×4.3 cm)	ECOG PS 2; PD-L1 3%; KRAS p.G12V+; MRI brain negative; adenocarcinoma on pleural cytology
September-November 2022	1st‑line: pemetrexed 750 mg + carboplatin 380 mg (cycles 1‑2), reduced to pemetrexed 700 mg + carboplatin 370 mg (cycle 3)	Restaging PET-CT (December 2022): objective radiological response (RECIST 1.1: partial response, confirmed by the treating clinician; formal measurements not available) with reduction in primary lesion metabolic activity and near-complete resolution of pleural effusion; residual active disease identified; METex14 skipping already known from baseline NGS	Fungal pneumonia after cycle 1; chemotherapy dose reduced. eGFR 75 mL/min/1.73 m²; creatinine 1.02 mg/dL
December 2022	2nd-line: capmatinib 200 mg BD initiated (biomarker-directed switch based on METex14 skipping mutation identified on baseline NGS; no documented progression)	ECOG deteriorated PS 2-3; multi-organ toxicity at ~6 weeks: blood glucose 350 mg/dL, creatinine 1.5 mg/dL, raised amylase/lipase, hypercalcemia, hyponatremia, 2 kg weight loss	Capmatinib discontinued ~February 2023
February 2023	3rd-line: tepotinib 225 mg OD initiated	Progressive clinical and biochemical improvement over 2 months; creatinine, amylase, and lipase trending toward normalization	Drug well tolerated; ECOG improved
April 2023	Grade 3 bilateral pedal edema detected; Doppler USS both limbs	DVT excluded; edema attributed to tepotinib. Schedule modified: 225 mg alternate-day; torsemide 5 mg BD added	-
May 2023	Grade 2 residual pedal edema; torsemide escalated to 20 mg OD	Tepotinib schedule adjusted to Monday-Friday (5 days/week, 2-day weekend holiday); calcium-restricted diet instituted; amylase/lipase fully normalized; weight and edema improving	-
June 2023	Restaging FDG PET-CT	Marginal reduction in primary lesion metabolic activity; significant nodal regression; pleural effusion resolved	Consistent with initial treatment response
October 2023	Interim FDG PET-CT	Apparent increase in radiological burden managed as suspected pseudoprogression; clinical and biochemical status stable	Tepotinib continued without interruption
February 2024	Constitutional symptoms: fatigue, anorexia, mild weight loss; creatinine 1.7 mg/dL	Tepotinib further reduced to 225 mg three days/week (Monday, Wednesday, Friday) for 2 weeks pending restaging; torsemide reduced back to 10 mg OD	13 months of continuous tepotinib therapy at this point
February 2024	Definitive restaging FDG PET-CT	Complete metabolic response; imaging criteria for complete remission met	Patient remains in complete remission at follow-up

## Discussion

This case provides clinically relevant insights into two aspects: First, intolerance to one MET TKI does not preclude benefit from another agent of the same class. Second, when standard dose reductions are exhausted, dose-frequency modification can control class-effect toxicity while preserving oncological efficacy.

The severe multi-organ toxicity seen with capmatinib in this patient, characterized by grade 3 hyperglycemia, acute kidney injury, pancreatic enzyme elevation, hypercalcemia, and hyponatremia, represents an uncommon but clinically significant toxicity profile [11]. The rapid normalization of all biochemical parameters upon capmatinib discontinuation in our patient supports a causal drug-toxicity relationship. Importantly, the successful transition to tepotinib shows that intolerance to one MET TKI does not preclude benefit from another agent of the same class. This finding aligns with the distinct pharmacologic profiles of these agents: an in vitro study found that tepotinib, unlike capmatinib, experiences reversible lysosomal retention, functioning as an intracellular drug reservoir that facilitates sustained MET receptor occupancy under fluctuating physiological conditions [12]. Although these pharmacokinetic differences relate primarily to target engagement and efficacy rather than directly explaining differential toxicity, they underline that capmatinib and tepotinib are structurally and pharmacologically distinct molecules with different exposure and off-target profiles, providing a plausible basis for agent-specific, rather than class-wide, intolerance as observed in our patient. The 2026 ASCO Living Guideline acknowledges that the decision between capmatinib and tepotinib may be guided by comorbidities and toxicity management requirements, supporting the clinical rationale for sequential MET TKI switching in the setting of agent-specific intolerance rather than class intolerance [13].

Peripheral edema is the hallmark class-effect toxicity of MET inhibitors, shown in 47-51% of patients receiving capmatinib and 63-70% of patients receiving tepotinib [5]. The suggested mechanism involves disruption of protective HGF/MET signaling in the vascular endothelium, increasing endothelial permeability and interstitial fluid accumulation [14,15]. The FDA-approved dose modification for tepotinib allows reduction from 450 mg to 225 mg once daily, with permanent discontinuation recommended if the reduced dose is not tolerated [16]. In our case, the patient was already getting 225 mg, leaving no further standard dose-reduction option. The treating clinician therefore implemented a novel stepwise dose-frequency reduction, from daily to alternate-day, then five days per week, and ultimately three days per week in conjunction with torsemide diuretic therapy. A recent multicentric Japanese real-world analysis confirmed the clinical importance of edema management: 71% of patients treated with tepotinib experienced edema, with grade ≥3 events in 12.9%; notably, patients who developed edema exhibited significantly longer progression-free survival than those without edema (10.8 months versus 4.2 months; hazard ratio: 0.31; p<0.001), suggesting that edema may be a pharmacodynamic marker of drug exposure and efficacy [17]. The co-occurrence of KRAS p.G12V with METex14 skipping in this patient is an uncommon molecular finding. The achievement of complete remission in the patient, despite the co-occurring KRAS mutation, indicates that the METex14 skipping alteration was the dominant oncogenic driver in this case. The apparent radiological progression in October 2023 was managed as suspected pseudoprogression on the basis of sustained clinical and biochemical stability. We acknowledge that pseudoprogression is uncommon with MET-targeted therapy and that this interpretation remains presumptive in the absence of histological confirmation or serial quantitative imaging; nevertheless, the subsequent complete metabolic remission supports the decision to continue treatment beyond apparent radiological progression under close clinical monitoring and underscores the importance of integrating clinical assessment with imaging findings.

## Conclusions

In this elderly, comorbid patient with METex14-mutated NSCLC, sequential tepotinib after capmatinib intolerance achieved complete metabolic remission. Tepotinib may therefore be considered after capmatinib intolerance when discontinuation is toxicity-driven rather than progression-related. Once standard dose reduction was exhausted, stepwise dose-frequency modification with diuretic support controlled grade 3 peripheral edema without apparent loss of efficacy. These single-patient observations support individualized MET inhibitor dosing in elderly, comorbid patients and warrant confirmation in larger real-world series.
